# Is Marathon Training Harder than the Ironman Training? An ECO-method Comparison

**DOI:** 10.3389/fphys.2017.00298

**Published:** 2017-05-29

**Authors:** Jonathan Esteve-Lanao, Diego Moreno-Pérez, Claudia A. Cardona, Eneko Larumbe-Zabala, Iker Muñoz, Sergio Sellés, Roberto Cejuela

**Affiliations:** ^1^All In Your Mind TMMadrid, España; ^2^Fundamentos de los Deportes, Universidad Europea de MadridMadrid, España; ^3^Departamento de Fisioterapia, Universidad del Valle de MéxicoMérida, Mexico; ^4^Texas Tech University Health Sciences CenterLubbock, TX, USA; ^5^Ciencias de la Salud, Universidad Europea del AtlánticoSantander, España; ^6^Departmental Section of Physical Education and Sports, University of AlicanteAlicante, Spain

**Keywords:** training intensity distribution, polarized training, training load quantification, endurance training, marathon, ironman

## Abstract

**Purpose:** To compare the absolute and relative training load of the Marathon (42k) and the Ironman (IM) training in recreational trained athletes.

**Methods:** Fifteen Marathoners and Fifteen Triathletes participated in the study. Their performance level was the same relative to the sex's absolute winner at the race. No differences were presented neither in age, nor in body weight, height, BMI, running VO_2max_ max, or endurance training experience (*p* > 0.05). They all trained systematically for their respective event (IM or 42k). Daily training load was recorded in a training log, and the last 16 weeks were compared. Before this, gas exchange and lactate metabolic tests were conducted in order to set individual training zones. The Objective Load Scale (ECOs) training load quantification method was applied. Differences between IM and 42k athletes' outcomes were assessed using Student's test and significance level was set at *p* < 0.05.

**Results:** As expected, Competition Time was significantly different (IM 11 h 45 min ± 1 h 54 min vs. 42k 3 h 6 min ± 28 min, *p* < 0.001). Similarly, Training Weekly Avg Time (IM 12.9 h ± 2.6 vs. 42k 5.2 ± 0.9), and Average Weekly ECOs (IM 834 ± 171 vs. 42k 526 ± 118) were significantly higher in IM (*p* < 0.001). However, the Ratio between Training Load and Training Time was superior for 42k runners when comparing ECOs (IM 65.8 ± 11.8 vs. 42k 99.3 ± 6.8) (*p* < 0.001). Finally, all ratios between training time or load vs. Competition Time were superior for 42k (*p* < 0.001) (Training Time/Race Time: IM 1.1 ± 0.3 vs. 42k 1.7 ± 0.5), (ECOs Training Load/Race Time: IM 1.2 ± 0.3 vs. 42k 2.9 ± 1.0).

**Conclusions:** In spite of IM athletes' superior training time and total or weekly training load, when comparing the ratios between training load and training time, and training time or training load vs. competition time, the preparation of a 42k showed to be harder.

## Introduction

The interest of recreational athletes in long distance events has been constantly growing in the last 30 years. As a sample of this, the world's marathon majors circuit reached about 200,000 runners in 2015 (WMM, 2016), while in triathlon, the Ironman® corporation events reached about 70,000 (WTC, 2016). The Ironman (IM) and Marathon (42k) distances are the classical longest endurance events in their respective sports. These events require large amounts of training (O'Brien et al., 1993; Laursen and Rhodes, 2001), so monitoring the training load becomes a must in order to prevent over reaching or overtraining (Halson, 2014).

“Training Load” (or training *stimulus*) implies the combination of the mode of exercise and the dose of the volume, intensity, and density or frequency (Wenger and Bell, 1986; Bompa and Haff, 2009). Quantifying the training load becomes of key importance, since it helps to consider the real demands of a given sport discipline (Bompa and Haff, 2009). There are many studies describing the physiological demands of a long distance event competition (Föhrenbach et al., 1987; O'Brien et al., 1993; Laursen and Rhodes, 2001), but few studies focused on the training load leading to a given performance (Esteve-Lanao et al., 2007; Guellich and Seiler, 2010; Seiler, 2010; Neal et al., 2013; Muñoz et al., 2014a,b; Stöggl and Sperlich, 2014). Bearing in mind that it is difficult to establish the precise amount of training load that an athlete needs (Seiler and Tønnessen, 2009), several studies have been focused on training intensity distribution between professional and recreational athletes including different disciplines (Robinson et al., 1991; Lucía et al., 2000, 2003; Billat et al., 2001; Esteve-Lanao et al., 2005; Seiler and Kjerland, 2006; Guellich et al., 2009). Different methods have been proposed to quantify training load (Borresen and Lambert, 2009). One of the few methods (if any) that allow the comparisons of the training load between different modes of exercise (i.e., running vs. swimming or cycling) is the Objective Load Scale (ECO in Spanish) method (Cejuela and Esteve-Lanao, 2011).

Since there are no data comparing athletes of different sports who perform at similar level for IM and 42k (i.e., trained recreational athletes), and starting from the simplistic athletes' question of “how hard it is” to be ready for these challenges, the goals of our study were: (1) To observe the differences between IM and 42k training load and (2) To observe the correlation between the competition time and the training intensity distribution at each group of endurance athletes.

## Methods

### Participants

Thirty recreational level athletes (15 long distance triathletes and 15 marathon runners) participated in the study. Both groups had 13 male and 2 females. They all volunteered and gave written informed consent to participate in the study, which had been approved by the Universidad Europea Ethical Advisory Committee. They all lived and trained in Spain, with the same coach (J. E-L). Their main goal for the season was to perform their best at an Ironman distance triathlon (3.8k swim, 180k cycle, and 42.1k run) or a Marathon (42.195k) race. Training and competitive experience in endurance sports was similar between subjects (~7 years). Subjects' descriptive characteristics are shown in Table 1.

**Table 1:** **Samplee characteristics**.

	**Ironman (*n* = 15)**	**Marathon (*n* = 15)**	***t*_28_**	***p***
Age (years)	39.1 (7.5)	38.2 (7.6)	0.34	0.737
Weight (kg)	70.9 (8.3)	69.7 (8.1)	0.42	0.675
Height (cm)	174.5 (7.3)	175.2 (5)	−0.32	0.751
BMI (kg/m^2^)	23.3 (2.4)	22.7 (2.3)	0.71	0.482
VO_2max_ (ml/min/kg)	56.1 (6.1)	57.5 (6.8)	−0.61	0.550
Training experience (year)	7.1 (2.1)	7.3 (2.3)	−0.41	0.672
Relative performance (%)	140.4 (24.5)	143.7 (22.2)	−0.38	0.705
Competition time (min)	704.6 (113.6)	186.5 (27.9)	17.15	<0.001
Same sex's absolute winner time (min)	503.7 (33.7)	130.1 (4.9)	42.49	<0.001

*Relative performance was calculated dividing competition time by absolute winner's time, considering the sex of each participant*.

### Main characteristics of training and periodization

**Figure 1:**
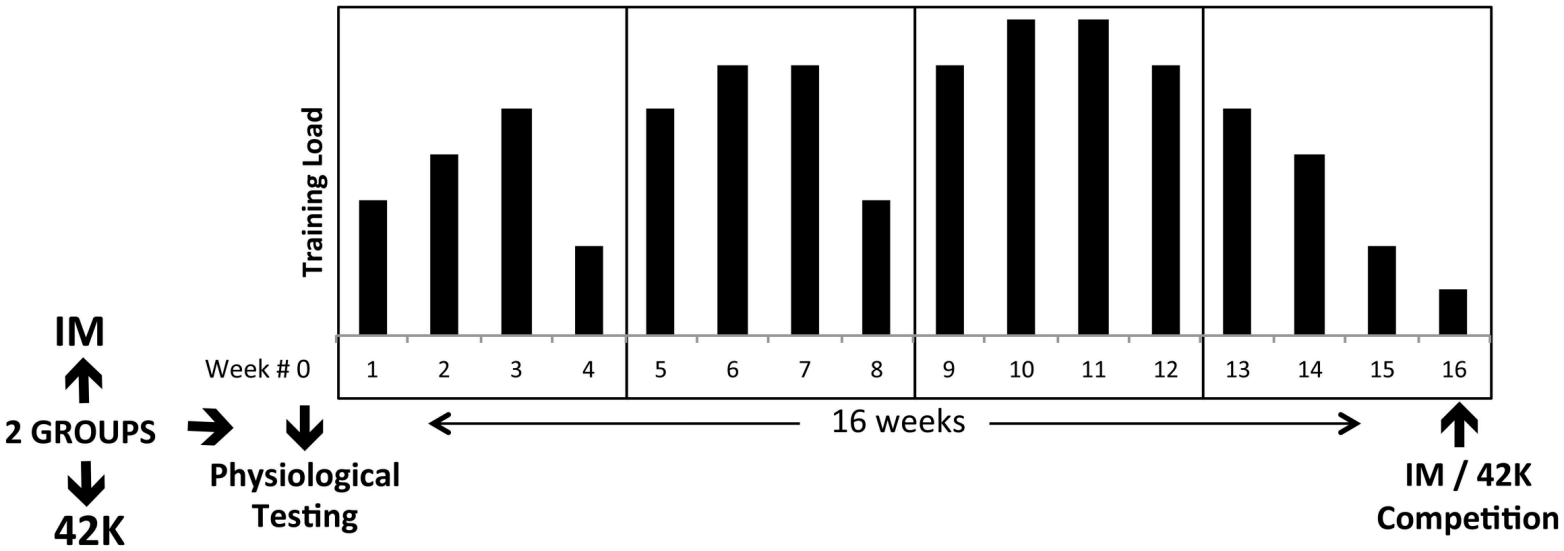
**Experimental design**. Planned training load is described with arbitrary units, with the purpose of showing general trend of hard/easy weeks.

Both 42k (runners) and IM (triathletes) groups completed a 16 weeks macrocycle (Figure 1). Global load was designed to alternate every 3 weeks of hard training load with an easy, lower load week (4, 4 weeks mesocycles). They followed an inverse periodization model, so that the peak training volumes were prescribed between weeks #10 and #11.

Hydration and nutritional guidelines were followed during these sessions, based on personal interviews with a sport nutrition specialist, including the calculation of the sweating rate. Time of day was set early in the morning for running or cycling sessions in order to minimize potential heart rate drift effects (Poole et al., 1994). Swimming sessions for triathletes were conducted twice to three times a week in the evening. Racing conditions were similar for both types of events (including temperature, humidity, altitude, and profile) and they were held in Spain during the spring season.

No differences were applied to the programs in strength training. This training was based on maximal strength development with moderate loads during the initial 11 weeks. It consisted in progressive workouts starting with resistance training machines. Loads progressed from 2 to 4 sets per muscle group, 25–8 reps, 40–75% of estimated 1RM through submaximal testing calculations. Those exercises were replaced at week 4 by multi-joint exercises. Loads were gradually increased in a similar fashion as mentioned before. Resistance training was gradually combined with specific strength methods in every sport (paddles for swimming, hills, or harder gears for cycling, light weighted belts for running) between weeks 5 and 13. By the end of the macrocycle, some basic maintenance resistance training on machines was conducted with moderate loads (60–70% of estimated 1RM), low number, explosive-velocity reps. Two sessions a week were programed (for most of the weeks with exception of the taper phase and the 4th weeks during each mesocycle). About 1.75 (0.20) sessions/week were reported. However, the training load for strength training was not quantified for this study.

No speed training or any other workouts beyond VO_2max_ zone were prescribed.

### Baseline physiological testing and training zones settings

Before starting the training program, all athletes participated in short-distance events macrocycles (10k or Half-Marathon for runners, Sprint or Olympic distance for triathletes), followed by a 3–4 weeks transition period. One week before starting the 16-week macrocycle, during that transition period, graded exercise tests were used to determine training zones.

Swimming tests were performed as a graded multi-stage test consisting of 7 repetitions of 200 m with 2 min rests (Pyne and Sweetenham, 2003). Heart rate (HR, beats·min^−1^) and blood lactate (bLA, mMol·L^−1^) samples from the earlobe were analyzed with a portable lactate analyzer (Lactate Pro, Arkray Inc, Amstelveen, NED). Triathletes were familiar with the Rating of Perceived Exertion (RPE) (Borg, 1998), so RPE 0–10 scale was applied immediately after collecting HR data and LA samples.

Threshold criteria were defined as follows: blood lactate 0.5 mMol·L^−1^ increase toward previous stage for Aerobic Threshold (AeT), >1.0 mMol·L^−1^ increase for Anaerobic Threshold (AnT), and 8–9 mMol·L^−1^ for Maximal Aerobic Velocity (MAV) (Beneke, 2003; Billat et al., 2003).

Cycling and running tests were conducted with a gas exchange analyzer (VO2000, Medical Graphics, St, Paul, MA, USA). A ramp-protocol test was conducted for cycling on an ergometer (Sensormedics, Yorba Linda, CA, USA) starting at 50 W and increasing 5 W every 12 s (Lucía et al., 2000). Two independent observers identified AeT and AnT. The following variables were measured: oxygen uptake (VO_2_), pulmonary ventilation (VE), ventilatory equivalents for oxygen (VE·${\text{VO}}_{2}^{-1}$), and carbon dioxide (VE·${\text{CO}}_{2}^{-1}$), and end-tidal partial pressure of oxygen (P_ET_O_2_) and carbon dioxide (P_ET_CO_2_).

Running tests were conducted on a treadmill (Technogym Run Race 1400 HC, Gambettola, Italy), with a starting velocity of 8 km·h^−1^, increased by 0.3 km·h^−1^ every 30 s until volitional exhaustion (Esteve-Lanao et al., 2007; Muñoz et al., 2014b).

For both cycling and running tests, standardized criteria were used for VO_2max_ achievement, AnT and AeT determination and HR recordings, following previously described procedures (Muñoz et al., 2014a,b).

### Training intensity distribution and training load quantification

Based on the classical 3-phase model of Skinner and McLellan (1980), and for practical purposes in terms of training intensity distribution analysis, three main zones were differentiated: <AeT (at or below AeT), BAeT-AnT (between thresholds, precisely beyond AeT and below AnT) and >AnT (at or beyond AnT). Of note, all data were included, both warm-ups and cool-downs, thus not following the so-called “Session Goal Aproach” applied in other studies (Seiler, 2010).

For daily training workouts, these three zones were subdivided into narrower ranges (dividing each zone for being more precise in some workouts, and adding a Maximal Aerobic Power zone for some swimming workouts), up to a total of 6 zones from <AeT to Maximal Aerobic Power plus 2 “anaerobic” zones. Microsoft® Excel® Training logs were designed to calculate training load based on the methodology of ECOs (Cejuela and Esteve-Lanao, 2011) which was specially developed for training quantification in triathlons, so that it is suitable for comparisons to any single event sport.

This methodology seems the most appropriate when comparing different endurance activities, as different exercise modes show different degrees of muscle damage, energy cost, effort densities, and differences at the ability of maintaining technique (Cejuela and Esteve-Lanao, 2011).

The Percentages of time spent at <AeT, BAeT-AnT, and >AnT were calculated by the training log dividing the total time spent during a workout at a given zone by the total exercising time during the workout and multiplied by 100. The training logs were prepared in order to record every session and to differentiate running or triathlon (swim/bike/run) sessions and calculations.

Briefly, the ECOs were calculated by multiplying the total duration of a training session (in minutes) with a scoring value between 1 and 50, depending on the heart rate-based training zone (1–8) and by a factor of 1.0, 0.75, or 0.5 for running, swimming or biking, respectively. Daily and weekly training loads (ECOs) of each subject were quantified. For example, a 60 min running session at Zone 1 is scored like this: 60 × 1 × 1, since Running has a factor of “1,” and Zone 1 has a factor of “1.” However, a 2-h cycling session at zone 1 also scores “60,” since Cycling has a factor of “0.5” (120 × 1 × 0.5). Another example (in the case of an interval training workout), would be a 12 × 100 m swimming workout at zone 4 plus other 20 min of zone 1 swimming adding the warm up and the cool down. If the swimmer would we performing at 1 min 40 s per rep, this would be a total of 20 min at zone 4. Thus, if the swimmer would we performing 20 (min) × Zone 1 is 20, and multiplied by 0.75 scores 15. When adding the other 20 min interval net time at Zone 4 per 0.75 is 60, so the total session scores 75 ECOs.

Both runners and triathletes were filling manually personal training logs with the information recorded in their HR monitors, considering net training time from the whole session, in terms of the amount of time spent per training zone at each sport (Cejuela and Esteve-Lanao, 2011).

Speed, Power or Pace values corresponding to the training zones were increased during the program according to RPE/HR initial training zones, as previously described (Muñoz et al., 2014a) and based on the reported validity of these lab references during a subsequent period of several months (Lucía et al., 2000). RPE was considered appropriate for technical swimming exercises and also when HR recording showed any anomalous display or abnormality.

Training Loads were designed to meet a mean (SD) of ~15,313(2,087) ECOs for the IM and 8,333(699) for 42k. The reason for this SD was that different programs were designed according to performance level differences, plus other considerations such as time availability or training experience. General training intensity distribution was scheduled week by week, with a global mean of 84/7/9%, respectively, in Zones 1/2/3 (IM 78/19/3, 42k 86/2/12).

Inclusion criteria were the following: (1) to complete 85% of total training sessions, (2) to record 95% of total training sessions, and (3) to complete and perform continuously, without any relevant health, tactical, or technical problems, the full distance in competition performing as best as possible.

### Data analysis

Training accomplishment was calculated dividing the training logs reported data by the prescribed training loads. Relative performance (%) was calculated dividing competition time by absolute winner's time, considering the sex of each participant, and multiplying the result by 100. Data were summarized as mean (standard deviation). Differences between IM and 42k athletes' outcomes were assessed using Student's *t*-test. Association between performance and training time and loads were assessed using Pearson's product-moment correlation coefficients. This statistical analysis was performed using SPSS version 22 (SPSS Inc., Chicago, Illinois, USA). The significance level was set at 0.05.

## Results

As shown in Table 1, IM and 42k athletes did not show any significant difference in age, weight, height, body mass index, nor VO_2max_. Additionally, their relative performance, compared to their sex's absolute winner, was also equivalent (40.4 and 43.7% over the winner's time, respectively). Mean performance time was 11 h 45 min for IM and 3 h 06 min for 42k.

Training logs were reported weekly and were filled by all the athletes. No relevant injuries, tactical or technical problems appeared along the training program or competitions. Based on total ECOs, an average of 98.7(20.2)% accomplishment was found.

**Figure 2:**
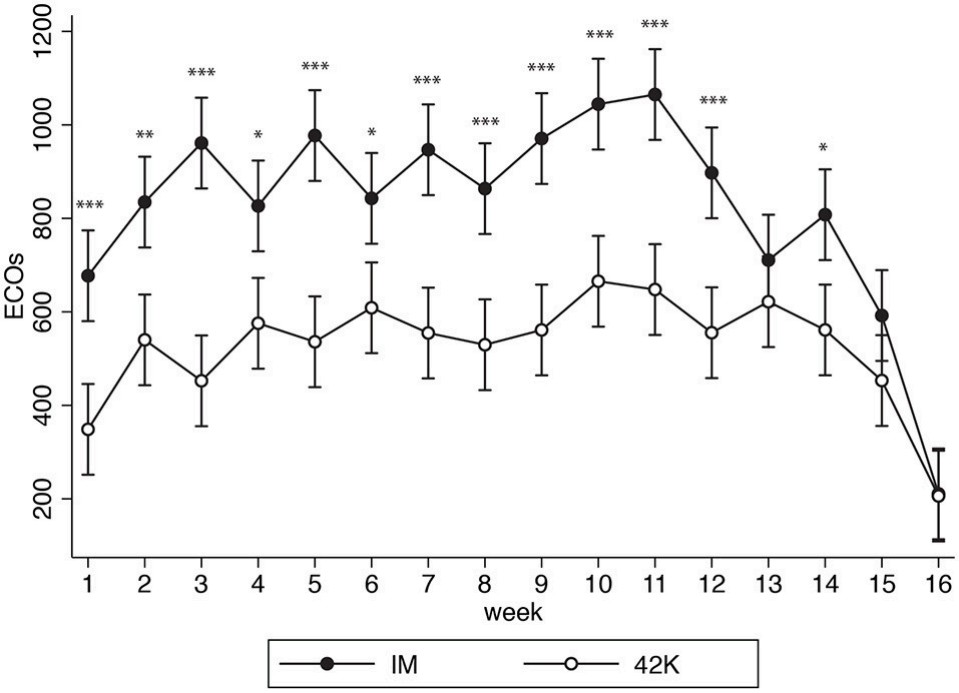
**Comparison of IM (black) and 42k (white) weekly training loads (ECOs) during 16 weeks**. ^*^p < 0.05; ^**^p < 0.01; ^***^p < 0.001.

As shown in Table 2 (or equivalently in Figure 2), IM athletes' loads were significantly higher during the greater part of training cycle. IM athletes invested significantly more time and ended up at higher loads, with higher weekly averages. Training peaks in time and loads were also significantly higher for IM athletes (Table 3).

**Table 2:** **Training Load per week (ECOs, weeks 1–16)**.

**Week (#)**	**Ironman (*n* = 15)**	**Marathon (*n* = 15)**	***t*_28_**	***p***
1	677.2 (169.4)	348.6 (162.5)	5.42	<0.001
2	834.8 (163.6)	540 (146.7)	5.20	<0.001
3	961.1 (276)	452.4 (190.7)	5.87	<0.001
4	826.6 (260.3)	575.5 (143)	3.27	0.003
5	977.1 (296)	535.9 (143)	5.20	<0.001
6	842.7 (251)	608.7 (143)	3.14	0.005
7	946.7 (404.9)	554.7 (136.8)	3.55	0.002
8	863.5 (193.5)	529.6 (162.2)	5.12	<0.001
9	970.7 (310.2)	561.2 (185)	4.39	<0.001
10	1044.3 (381.3)	665.4 (218.5)	3.34	0.002
11	1064.9 (400.8)	647.7 (227.5)	3.51	0.002
12	897.3 (248.9)	555.3 (225.2)	3.95	<0.001
13	710.7 (274.5)	621.7 (214.2)	0.99	0.331
14	807.8 (347.1)	561.3 (229.6)	2.30	0.031
15	592.1 (267.6)	453.0 (239.5)	1.50	0.145
16	210.5 (160.8)	206.0 (72.8)	0.10	0.923

**Table 3:** **Load description as total values, weekly averages, percentages of load by zone, peak values, and relative loads to training and competition time**.

	**Ironman (*n* = 15)**	**Marathon (*n* = 15)**	***t*_28_**	***p***
**TOTALS**				
Total training time (h)	206.7 (40.8)	84.3 (15.5)	10.86	<0.001
Total training load (ECOs)	13347.1 (2732.5)	8416.6 (1887.8)	5.75	<0.001
**WEEKLY AVERAGES**				
Training weekly avg time (h)	12.9 (2.6)	5.2 (0.9)	10.90	<0.001
Training weekly avg time (min)	775.5 (153.7)	311.4 (56.2)	10.98	<0.001
Average weekly load (ECOs)	834.1 (170.7)	526 (118.1)	5.75	<0.001
**TRAINING BY ZONES**				
% of Time in zone <AeT	67.5 (13.6)	74.6 (3.9)	−1.94	0.070
% of Time in zone BAeT-AnT	28.4 (11.8)	15.6 (4.9)	3.87	0.001
% of Time in zone >AnT	4.3 (2.3)	9.7 (3.9)	−4.64	<0.001
% of Load in zone <AeT	45.8 (11.9)	48.3 (5)	−0.74	0.470
% of Load in zone BAeT-AnT	40.8 (9.8)	26.5 (8.1)	4.37	<0.001
% of Load in zone >AnT	13.7 (5.7)	25.1 (10.4)	−3.75	0.001
**PEAKS**				
Training time peak (h)	20.9 (4.8)	8.8 (1.8)	9.17	<0.001
Peak load (ECOs)	1345.5 (355.1)	838.1 (178.9)	4.94	<0.001
Peak load week (#)	7.9 (1.9)	6.5 (2.7)	1.63	0.115
**RATIOS BY TRAINING TIME**				
Training load (ECOs)	65.8 (11.8)	99.3 (6.8)	−9.50	<0.001
**RATIOS BY COMPETITION TIME**				
Training time (h)	1.1 (0.3)	1.7 (0.5)	−3.99	<0.001
Training load (ECOs)	1.2 (0.3)	2.9 (1)	−6.69	<0.001

Peak Training Load was located about the same period from competition in both groups (between 6th and 8th before competition). During the last 4 weeks, the loads were reduced in both groups, but more markedly in IM, so that no differences were found during that last mesocycle.

Although no significant differences were found in relative time and load in zone <AeT, IM athletes showed significantly higher relative times and loads in zone BAeT-AnT, while 42k runners' relative time and load in zone >AnT were significantly higher (Table 3).

Ratios of training load by training time in 42k runners showed significantly higher relative training load [*t*_(28)_ = −9.50, *p* < 0.001]. Additionally, ratios of training time [*t*_(28)_ = −3.99, *p* < 0.001] and load relative to competition time [*t*_(28)_ = −6.69, *p* < 0.001] also favored 42k runners.

When observing correlations with performance time, both groups showed significant, inversely related correlations between total training time and performance (the more training time, the better performance). However, only the 42k group showed large and significant correlation between total training load and performance.

Relative time and loads in zone <AeT showed a negative association to performance only for IM, but not for 42k (where only time in Zone <AeT was significantly related). Conversely, absolute and relative time in zone >AnT, and relative load in zone >AnT, showed a negative association to relative performance only for 42k, but not for IM. Relative time and loads in zone BAeT-AnT showed a significant association with poor performance for both groups (so more training time or load BAeT-AnT, the worst performance). Total training time in Zone BAeT-AnT did not show significant associations (see Table 4).

**Table 4:** **Correlation coefficients (and ***p***-value) between performance (min) and training load distribution**.

	**IM**	**42k**
**TOTAL**		
Total training time (h)	−0.59 (0.021)	−0.80 (<0.001)
Total training load (ECOs)	−0.04 (0.894)	−0.73 (0.002)
**ZONE** <**AeT**		
Training time in zone <AeT	−0.74 (0.002)	−0.82 (<0.001)
% of Time in zone <AeT	−0.70 (0.004)	−0.10 (0.723)
% of Load in zone <AeT	−0.60 (0.019)	0.37 (0.175)
**ZONE BAeT-AnT**		
Training Time in zone BAeT-AnT	0.47 (0.075)	0.11 (0.700)
% of Time in zone BAeT-AnT	0.71 (0.003)	0.65 (0.009)
% of Load in zone BAeT-AnT	0.54 (0.038)	0.79 (<0.001)
**ZONE** >**AnT**		
Training time in zone >AnT	0.33 (0.223)	−0.77 (0.001)
% of Time in zone >AnT	0.44 (0.099)	−0.74 (0.002)
% of Load in zone >AnT	0.33 (0.234)	−0.81 (<0.001)

## Discussion

Both groups presented a significant association between more <AeT training and better performance. However, the opposite association was found with BAeT-AnT, so that the more you train “moderate,” the worse in IM or 42k. This agrees with previous studies conducted with age-group IM triathletes (Muñoz et al., 2014a), recreational 10k runners (Muñoz et al., 2014b), and trained Cross Country runners (Esteve-Lanao et al., 2005, 2007). To our knowledge, this is the first study to identify these associations in recreational trained marathon runners.

Interestingly, >AnT was associated to better performance in 42k but not in IM. According to the existing literature (O'Brien et al., 1993; Laursen and Rhodes, 2001), we suspect that physiological intensity differences between these two events might explain this. In contrast with the opposite pattern shown between performance and “easy” (<AeT) or “moderate” (BAeT-AnT) zones time or load, “intense” >AnT zone seems to be linked to a better 42k performance, but not in IM. Again, the superior intensity which marathoners perform seems to explain why this association might be found. For instance, a 3 h marathoner (like those in our study) will average around 70% of VO_2max_ during the race (O'Brien et al., 1993), which would be BAeT-AnT zone in our runners' assessments. However, only during the swimming it might be reasonable to exert beyond AeT (and maybe at some particular moments on the bike) during an IM distance triathlon (Muñoz et al., 2014a). Thus, this opens new insights for the general assumption of the benefits of “Polarized Training Distribution,” so that it might not be “always better” for all disciplines. In fact, previous studies reported polarized distribution in top elite marathoners (Billat et al., 2001), but not in recreational IM distance triathletes (Neal et al., 2011; Muñoz et al., 2014a).

The training intensity distribution found in this study (considering both groups) is in line with other sports (Billat et al., 2001; Lucía et al., 2003; Fiskerstrand and Seiler, 2004; Seiler and Kjerland, 2006; Guellich and Seiler, 2010). One of the more active groups of researchers in this issue is Seiler's (Seiler and Kjerland, 2006; Seiler and Tønnessen, 2009; Seiler, 2010) who pointed out a 75-5-20 or even 80-0-20 distribution as “optimal,” which seems to be really difficult to achieve unless discarding the warm-ups and cool-downs. Had we done it, we would have probably reached superior >AnT zone percentage. However, as previously described, since they were recreational athletes (i.e., reduced time and training frequency) the training programs increased the weekly time spent in easy zones by using relatively long warm-ups and cool-downs.

Load dynamics were not exactly as originally designed. Peak load week was approximately the same between groups, although tapering was different. In any case, the tapering technique was progressive, particularly in 42k. As previously discussed in the literature, progressive, non-linear tapering techniques seem to have a more pronounced positive impact on performance than step-taper strategies (Mujika and Padilla, 2003).

As suspected, IM athletes trained more than 42k runners. Particularly, IM group trained about more than twice in time and about 1/3 in training load. Recently, it was reported that national level triathletes trained an average of 1,256 ECOs per week over the same period of time as our study (unpublished data from Saugy et al., 2016). Triathletes in our study averaged 834. Top Class Marathoners (according to our calculations using the ECOs method), were 1,200 ECOs whilst trained marathoners would be around 1,000 weekly (Billat et al., 2001). The marathoners in our study averaged 526 ECOs. These and future data might help general references toward optimal doses in relation to performance level, sports and events, although it is known that training response and performance outputs are multifactorial, including genetics (Smith, 2003).

The original question of this study was about “how hard it is” to train for an IM vs. for a 42k. The approach that has been conducted compares training time and load in relation to competition. For instance, classical swimming volumes are tremendously high compared to the competition distance (Mujika et al., 1995). The findings of our study state that 42k training is harder in relation to the competition demands. Training Load per training hour is significantly higher (99.3 ECOs in 42k vs. 65.8 in IM), which is about 1.5 ECOs in 42k training, vs. 1 ECO per min in IM. Moreover, Average Weekly Training Load per every minute spent in competition is higher for 42k (2.9 vs. 1.2), as well as the training time invested per every minute in competition (1.7 vs. 1.1, 42k vs. IM). This is the first study to compare athletes from similar level. Further studies will have to present new comparisons in terms of performance level, sports and disciplines.

The main limitation in our study was that it was conducted with athletes who were trained by the same coach, so the applicability of the results should be restricted to similar conditions. This was done because it allowed a better control of many aspects of the program (such as baseline loads, mesocycles distributions, program length, peak volume location, taper design, strength, and speed training methodology, and training supervision). General training intensity distribution was scheduled to be ~84/7/9%, respectively, in Zones 1/2/3. However, it showed a global mean of ~72/20/8%, respectively, in Zones 1/2/3. IM should be ~78/19/3% but it was ~68/28/4%, 42k should be ~86/2/12%, but it was ~74/16/10%. This training intensity distribution has been classified as “pyramidal” (Stöggl and Sperlich, 2015). According to Zone 1 and Zone 2 distributions, both showed a standard deviation of ~11% among all athletes and both groups. Consequently, in spite of sharing the same coach supervision, a high variability between programs' loads, accomplishment and intensity distribution were given.

The ECOs method has been recently used in a dozen of peer-reviewed papers with elite and high level athletes (Debevec et al., 2015; Hauser et al., 2016; Saugy et al., 2016; Villaño et al., 2016). Further studies should focus on training tolerance between different athletes' disciplines but including biomarkers to relate these theoretical training load comparisons and discriminate between hormonal status, muscle damage, oxidative stress levels or others.

## Conclusions

The highest associations between performance and training were found with time and % of time in zone <AeT for IM, whilst in 42k it was related to the zone <AeT time or load, plus the accumulation of any variable related to >AnT.

In spite of IM athletes' superior training time and total or weekly training load, and according to the ratios between training load and training time, and training time or training load vs. competition time, the preparation of a 42k showed to be harder.

## Ethics statement

This study was carried out in accordance with the recommendations of the Ethics Committee of the Universidad Europea de Madrid, with written informed consent from all subjects. All subjects gave written informed consent in accordance with the Declaration of Helsinki. The protocol was approved by the Ethics Committee of the Universidad Europea de Madrid.

## Author contributions

JE and RC made the general design. JE trained the athletes. JE, RC, IM, and EL wrote the manuscript. DM and CC participated in the data acquisition conducting the performance tests, supervising the training workouts, supplying on line assistance and preparing the data. EL analyzed the data and wrote a substantial part of the results section. SS reviewed the manuscript and made important contributions to the final edition.

### Conflict of interest statement

The authors declare that the research was conducted in the absence of any commercial or financial relationships that could be construed as a potential conflict of interest.
